# Peripheral Nerve Stimulator for Pain After Surgery for Ulnar Neuropathy at the Elbow

**DOI:** 10.7759/cureus.37297

**Published:** 2023-04-08

**Authors:** Casandra Mysior, Nicole Walch, Akshat Gargya

**Affiliations:** 1 Department of Anesthesiology, University of Vermont Medical Center, Burlington, USA; 2 Department of Anesthesiology and Pain Management, The Robert Larner, M.D. College of Medicine at The University of Vermont, Burlington, USA

**Keywords:** ultrasound, neuromodulation, peripheral nerve stimulator, neuropathic pain syndrome, ulnar nerve neuropathy

## Abstract

Neuropathy secondary to ulnar nerve entrapment is a painful condition that often persists following surgical decompression. We present the case of a 43-year-old female with Ehlers-Danlos syndrome and left ulnar neuritis refractory to surgical management. Peripheral nerve stimulation of the ulnar nerve proximal to the elbow resulted in a significant reduction in pain and improvement in disability post-implantation. This case suggests that peripheral nerve stimulation is a promising minimally invasive technique that should be considered for treating non-operative upper extremity neuropathic pain.

## Introduction

Ulnar nerve entrapment is one of the most common nerve entrapments in the upper extremity, second only to carpal tunnel syndrome [1]. Patients commonly present with hypesthesia in an ulnar nerve distribution accompanied by pain at the elbow radiating to the fourth and fifth digits [2]. Pain increases with repetitive elbow extension and flexion, making it difficult for the patient to perform their activities of daily living. If continued for an extended period, patients can experience muscle atrophy and weakness [2]. Timely surgical management has a higher success rate in reducing mild, intermittent sensory and motor deficits. Patients who experience prolonged symptoms tend to have less favorable surgical outcomes. However, more than 50% of patients undergoing surgical decompression, irrespective of technique, will experience continued pain and paresthesia [3]. With a lack of clear management protocols and currently available treatment options, the care of patients with failed ulnar decompressive surgery poses an enormous challenge for orthopedic specialists. Our case report describes treating a patient with chronic pain after ulnar decompressive surgery with an ulnar nerve peripheral nerve stimulator (PNS).

## Case presentation

Our patient is a 43-year-old female with a past medical history significant for Ehlers-Danlos syndrome (EDS) with recurrent left ulnar neuritis. She underwent cubital tunnel release and ulnar nerve transposition 10 years before the presentation and reported significant pain reduction from the surgery. However, two years before presentation, the patient noticed increased pain in a left ulnar nerve distribution and a palpable mass at her left elbow. The mass was revealed to be a herniation of the flexor carpi ulnaris muscle, causing left ulnar nerve compression. The fascial defect was repaired by orthopedic surgery, providing her with acute pain relief. However, the herniation recurred after three months, and her symptoms returned. Due to impaired tissue quality in the setting of EDS, the Plastic and Orthopedic Surgery teams considered using allograft to repair the fascial defect but remained concerned about the risk of graft failure; hence, surgical management was deferred. The patient was then seen in the Pain Management clinic. At the presentation, she described her pain as originating at the left medial elbow and radiating to the fourth and fifth digits. The pain was 4 on the Numeric Pain Rating Scale (NPRS), described as sharp, shooting, burning, and tingling, severely limiting her function. It was exacerbated by elbow flexion, typing, and direct pressure over the medial elbow and forearm. Her physical exam was notable for decreased sensation to light touch and allodynia from above the medial epicondyle to the ulnar fingers. The range of motion of the cervical spine, elbow, wrist, and fingers was unrestricted. Strength was intact in finger flexors, extensors, abductors, and wrist and elbow flexors and extensors. Grip strength was grossly symmetric. The Spurling maneuver was negative. Tinel sign was positive over the transposed ulnar nerve (localized with ultrasound) at the distal aspect of the surgical incision over the FCU muscle and at Guyon’s canal. Evaluation with the Disabilities of the Arm, Shoulder, and Hand questionnaire (DASH) revealed a disability/symptom score of 82.5% [4]. Her Leeds Assessment of Neuropathic Symptoms and Signs Pain Scale (LANSS) was 19/24, which signified neuropathic mechanisms contributing to her pain [5]. Electrodiagnostic testing of the left upper extremity, including inching along the ulnar nerve at the elbow, was normal. MRI of the cervical spine was notable for multilevel degenerative disc and facet disease, superior endplate bone edema with slight loss of vertebral body height at C5 and C6, considered to be degenerative in nature, moderate-to-severe left and mild-to-moderate right foraminal stenosis at C6/7, and mild-to-moderate left neural foraminal stenosis at C5/6 levels. A computerized tomography angiogram of the neck and subclavian arteries was normal. In addition to surgical interventions, prior treatment efforts included physical therapy and aquatic physical therapy with an ongoing home exercise program which were ineffective in reducing her pain. She was prescribed gabapentin at a total daily dose of 2100 mg, amitriptyline 100 mg at bedtime, topical lidocaine 5% patches, and nonsteroidal anti-inflammatory medications as needed. She had a local anesthetic and steroid injection that did not provide durable relief.

**Figure 1:**
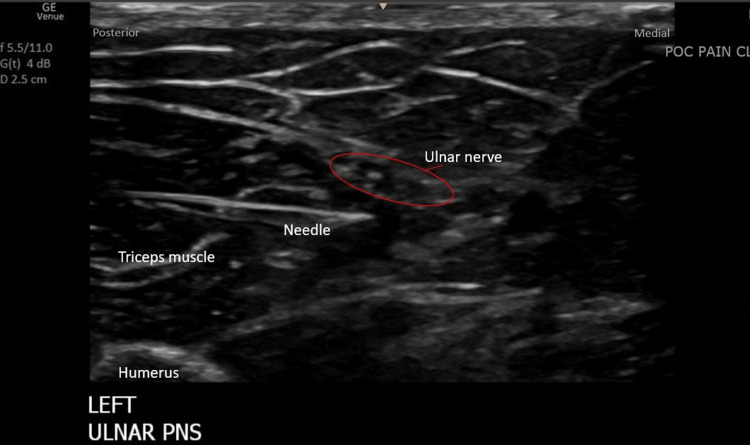
Ultrasound image using a linear array transducer on the posterolateral side of the arm demonstrating the procedural anatomy with the location of final lead placement.

The patient underwent a successful uncomplicated left ulnar nerve PNS placement in the pain clinic. The percutaneous PNS lead was implanted under ultrasound guidance targeting the ulnar nerve approximately 10 cm above the medial epicondyle in a short axis, posterior to the medial approach through the triceps muscle (Figure 1).

The lead tip (MicroLead; SPR Therapeutics, Cleveland, Ohio, USA) was implanted approximately 0.25 cm from the nerve to enable selective activation of large-diameter sensory fibers. Post-procedurally, the patient reported 100% improvement in her left ulnar arm pain and had a pain score of 0/10. At two months, she continued to experience a significant reduction in pain and improved function. Her pain score in the ulnar distribution remained 0/10 on the NPRS pain scale, and her DASH score fell from 82.5% to 38.3%, signifying significant improvement in disability. She continued to report improved physical function, sleep, and quality of life.

## Discussion

There are various surgical techniques to relieve ulnar nerve compression at the elbow, from simple decompression to medial epicondylectomy and various methods of anterior transposition [6]. Despite decades of clinical experience in early diagnosis and surgical techniques, chronic pain from ulnar entrapment remains challenging [7]. In our case, the patient’s past medical history of EDS predisposed her to neuropathic syndromes, affected surgical outcomes, and tempered expectations for the success of revision surgeries [8]. Pain management is extremely challenging in patients with refractory pain after initial ulnar entrapment release surgery, especially those who do not have secondary surgical treatment options. In our case study, we report the successful management of this debilitating refractory pain with a PNS, which led to a reduction in pain and an improvement in disability. Similar to diabetic mononeuropathies, the mechanism of local tenderness at the site of entrapment is most likely due to nerve trunk pain mediated by Nervi nervorum [9]. As per morphological studies done by Ochoa J and Verdugo RJ, entrapment lesions damage myelinated fibers only in severe lesions; C fibers are preserved in mild-to-moderate lesions [10]. The mechanism of radiation of pain is even more vaguely studied and explained. 

PNS is currently used in adults for various indications, including complex regional pain syndrome (CRPS), post-herpetic neuralgia, lumbar and thoracic spinal neuropathy, and peripheral neuropathic pain from the sciatic, femoral, or saphenous nerve [11-15]. PNS acts both on the peripheral and central nervous systems and modulates inflammatory pathways and the autonomic nervous system [16,17]. Imaging modalities, including positron emission tomography and functional MRI, have also demonstrated their role in the activation of critical cortical areas [17,18]. In our patient, we believe its effects on the inflammatory pathways and selective activation of sensory fibers causing inhibition of A-delta and C fibers in the peripheral nervous system helped reduce the patient’s chronic pain. A previous study by Bouche B et al. on 26 patients with chronic medically refractory neuropathic pain of the upper limb secondary to CRPS and nerve injury treated by brachial plexus or supra-scapular nerve PNS reported PNS to be a safe, minimally invasive, and an effective treatment option to control upper limb neuropathic pain [19]. There is currently one reported case where the patient experienced 33% pain reduction and substantial functional improvement after ulnar peripheral nerve stimulation after a failed ulnar transposition surgery [20]. Our case additionally includes a DASH questionnaire to quantify the improvement in disability. As per clinical guidelines from the American Society of Pain and Neuroscience for the use of implantable PNS, there is currently level II evidence to support the use of PNS for upper extremity pain and neuropathic syndrome [12]. Our case report further provides evidence to support its use for ulnar entrapment syndromes and upper extremity neuropathy. In case of suboptimal long-term effects after temporary PNS, a permanent PNS can be considered in this patient.

## Conclusions

In conclusion, our case report describes the successful management of a patient with chronic pain with ulnar nerve PNS after unsuccessful pain control from surgical management of ulnar neuropathy at the elbow. Our case report provides a new indication for the use of PNS in the management of chronic pain. It will help draw future consensus guidelines for pain management in patients with ulnar neuropathy/entrapment/nerve compression syndromes. Further studies, especially prospective research trials, are required to provide more evidence of its role after surgical management has failed to provide sufficient pain relief. 

## References

[1] Mangi MD, Zadow S, Lim W (2022). Nerve entrapment syndromes of the upper limb: a pictorial review. Insights Imaging.

[2] Cambon-Binder A (2021). Ulnar neuropathy at the elbow. Orthop Traumatol Surg Res.

[3] Gabel GT, Amadio PC (1990). Reoperation for failed decompression of the ulnar nerve in the region of the elbow. J Bone Joint Surg Am.

[4] Gummesson C, Atroshi I, Ekdahl C (2003). The disabilities of the arm, shoulder and hand (DASH) outcome questionnaire: longitudinal construct validity and measuring self-rated health change after surgery. BMC Musculoskelet Disord.

[5] Bennett M (2001). The LANSS Pain Scale: the Leeds assessment of neuropathic symptoms and signs. Pain.

[6] Mackinnon S, Dellon A (1988). Ulnar nerve entrapment at the elbow. Surgery of the Peripheral Nerve.

[7] Antoniadis G, Richter HP (1997). Pain after surgery for ulnar neuropathy at the elbow: a continuing challenge. Neurosurgery.

[8] Fernandez A, Aubry-Rozier B, Vautey M, Berna C, Suter MR (2022). Small fiber neuropathy in hypermobile Ehlers Danlos syndrome/hypermobility spectrum disorder. J Intern Med.

[9] Scadding JW (2013). Peripheral neuropathies. Textbook of Pain.

[10] Ochoa J, Verdugo RJ (2001). Mechanisms of neuropathic pain: nerve, brain, and psyche: perhaps the dorsal horn but not the sympathetic system. Clin Auton Res.

[11] Mainkar O, Singh H, Gargya A, Lee J, Valimahomed A, Gulati A (2021). Ultrasound-guided peripheral nerve stimulation of cervical, thoracic, and lumbar spinal nerves for dermatomal pain: a case series. Neuromodulation.

[12] Strand N, D'Souza RS, Hagedorn JM (2022). Evidence-based clinical guidelines from the American Society of Pain and Neuroscience for the use of implantable peripheral nerve stimulation in the treatment of chronic pain. J Pain Res.

[13] Gargya A, Singh H, Lin T, Gulati A (2020). Extraforaminal thoracic and lumbar spinal nerve ultrasound-guided percutaneous peripheral nerve stimulation. Pain Med.

[14] Singh H, Gargya A, Lin T, Gulati A (2020). Sciatic, femoral, and lateral femoral cutaneous nerve ultrasound-guided percutaneous peripheral nerve stimulation. Pain Med.

[15] Fritz AV, Ferreira-Dos-Santos G, Hurdle MF, Clendenen S (2019). Ultrasound-guided percutaneous peripheral nerve stimulation for the treatment of complex regional pain syndrome type 1 following a crush injury to the fifth digit: a rare case report. Cureus.

[16] Plazier M, Vanneste S, Dekelver I, Thimineur M, De Ridder D (2011). Peripheral nerve stimulation for fibromyalgia. Prog Neurol Surg.

[17] Lin T, Gargya A, Singh H, Sivanesan E, Gulati A (2020). Mechanism of peripheral nerve stimulation in chronic pain. Pain Med.

[18] Bandeira JS, Antunes LD, Soldatelli MD, Sato JR, Fregni F, Caumo W (2019). Functional spectroscopy mapping of pain processing cortical areas during non-painful peripheral electrical stimulation of the accessory spinal nerve. Front Hum Neurosci.

[19] Bouche B, Manfiotto M, Rigoard P, Lemarie J, Dix-Neuf V, Lanteri-Minet M, Fontaine D (2017). Peripheral nerve stimulation of brachial plexus nerve roots and supra-scapular nerve for chronic refractory neuropathic pain of the upper limb. Neuromodulation.

[20] Langford B, D'Souza RS, Pingree M, Mauck WD (2022). Treatment of ulnar neuropathic pain with peripheral nerve stimulation: two case reports. Pain Med.

